# Tribological Properties of Composites Based on Single-Component Powdered Epoxy Matrix Filled with Graphite

**DOI:** 10.3390/ma17133054

**Published:** 2024-06-21

**Authors:** Jakub Smoleń, Krzysztof Stępień, Marta Mikuśkiewicz, Hanna Myalska-Głowacka, Mateusz Kozioł, Marcin Godzierz, Henryk Janeczek, Jan Czakiert

**Affiliations:** 1 Faculty of Materials Engineering, Silesian University of Technology, Krasińskiego 8, 40-019 Katowice, Poland; 2 Centre of Polymer and Carbon Materials, Polish Academy of Sciences, M. Curie-Skłodowskiej 34 Street, 41-819 Zabrze, Poland; 3 DeGroote School of Business, McMaster University, 1280 Main St W, Hamilton, ON L8S 4L8, Canada

**Keywords:** polymer–matrix composite, tribology, self-lubricating materials, graphite, sliding materials

## Abstract

Composites based on powdered single-component epoxy matrix are an alternative technological solution for composites produced using liquid epoxy resins. This article describes in detail the process of producing graphite-reinforced composites for tribological applications. The advantages and disadvantages of technological processes where the matrix is a single-component epoxy powder were demonstrated, and the properties of the obtained materials were examined. A series of composite materials with the graphite filler with sizes below 10 μm and below 45 μm and weight additions of 5, 10, 20, 30% were produced. Mechanical tests and tribological tests conducted with the pin-on-block method were performed, and the mechanism of tribological wear was described. The conducted research allowed us to conclude that the incorporation of graphite, regardless of particle size, above 10% by weight results in a significant reduction in the friction coefficient (approximately 40–50% lower than in unfilled epoxy resin), which is beneficial in the production of cheap self-lubricating materials.

## 1. Introduction

The design and production of materials for tribological applications can be carried out either by maximizing or minimizing the coefficient of friction depending on the function of a given product. Materials with a high coefficient of friction are desirable for applications such as car brakes, where high values of the friction coefficient are more effective in operation [1]. Szymański et al. describe a review of metal composites used in the production of brake discs, paying particular attention to the economics of materials and production costs [2]. It is important that the development of materials and technologies alone is not always sufficient to implement the achievement in industrial conditions, because the costs are often too high for the customer, which forces designers and manufacturers to develop effective and cheap solutions. In the article by Posmyk and Mylaski [3], the authors developed friction composites intended for the production of brake discs and brake drums with more than three times lower abrasive wear thanks to the use of SiC foam reinforcement instead of SiC powder dispersion. Among the most important conclusions, the authors mentioned environmental benefits, where reduced abrasive wear contributes to less dust. Materials with low friction coefficient values are beneficial in the production of self-lubricating materials [4]. The authors acknowledge that self-lubricating composites are widely used in traditional and modern industrial sectors, such as automotive, aerospace, space, and materials processing due to the ability to work in very difficult conditions, such as high temperatures, high loads, high vacuum, chemically reactive environments, etc. Rodiouchkina et al. noticed that the abrasive wear of self-lubricating materials is related to the tendency to form a transfer film on the surface [5]. Rahmanian et al. studied the behavior of self-lubricating materials in space applications using specially developed test systems [6]. The authors of this publication paid particular attention to the fact that self-lubricating materials must maintain their lubricating properties for a long time without the possibility of maintenance. Regardless of the value of the friction coefficient, it is advantageous to obtain minimal abrasive wear, which is suitable for limited material consumption. Proper lubrication of wear materials enables optimization of sliding and wear characteristics. Therefore, production of self-lubricating materials is important for many industrial sectors.

The use of composites in the production of tribological materials is important due to the possibility of adapting the properties to the application by changing the composition implemented by the appropriate selection of the matrix and reinforcing phase. The use of polymer–matrix composites in tribology allows us to develop the potential of unfilled polymer material with the properties of additional materials. The production of composites for tribological applications is constantly growing due to the possibility of achieving more favorable properties than those of conventional homogenic materials [7]. The addition of reinforcement allows us to change the tribological properties, e.g., reducing the coefficient of friction and limiting abrasive wear, changing the mechanical properties, changing the thermal properties, etc. The addition of short fibers (glass, carbon, or other) to the polymer matrix increases the mechanical properties and improves the load-bearing capacity of the bearings [8,9]. The addition of solid lubricants in the form of powders mainly contributes to a reduction in the friction coefficient [10]. It is possible to produce hybrid composite structures for tribological applications, where short fibers increasing mechanical strength and powder fillers reducing the friction coefficient are added to the polymer matrix, which is currently a dynamically developed area in tribology [11,12,13].

Fillers (solid lubricants) that effectively reduce the friction coefficient of polymer composites have been described in numerous publications. The use of solid lubricants that form a transfer film on the surface of self-lubricating composites is beneficial and limits the use of liquid lubricants in use. Liquid lubricants require regeneration and disposal, which generates additional costs and constitutes an environmental burden [14]. Fillers with low friction resistance that are used to effectively reduce the friction coefficient of materials are soft metals (e.g., lead [15], copper, nickel [16]), soft polymers (e.g., polyamide [17], PTFE [18]), graphite [19,20], molybdenum disulfide [21,22], and others [23]. The most commonly used polymer materials for the production of sliding materials are polyamides [24], PTFE [25], polyamide imides [26], polyimides [27], polyethylenes [28,29], PEEK [30] phenol-formaldehyde resins [31,32], and epoxy resins [33,34,35].

Single-component epoxy powders are gaining popularity, and their industrial use is expanding to include new products. These systems are characterized by a convenient curing temperature and a short cross-linking time. They do not require separate components such as epoxy resin and a curing agent. One-component epoxy powders contain thermal latent curing agents that limit pollutants emitted into the environment (including volatile compounds) and ensure the requirements for large-scale production [36,37,38]. Previous scientific reports discussed the short (several days) stability of single-component epoxy powders at room temperature. Significant scientific and technological progress in recent years has led to stability up to 12 months at room temperature [39], so the main disadvantage of one-component systems is obsolete, and resin manufacturers are constantly extending the lifetime.

This article is a continuation of previous research conducted on self-lubricating epoxy composites with the addition of graphite fillers. The previous publication [40] discussed epoxy composites produced on the basis of typical liquid epoxy resins. In this publication, the liquid epoxy resin is replaced by a single-component epoxy in powder form. This modification requires a complete change in the technological process of material production but may have many benefits resulting from, among others, the possibility of automating the production process in mass production and the possibility of increasing the percentage of addition of fillers (graphite). A set of composites was produced with varying amounts of graphite addition from 5% to 30% by weight for two sizes of graphite (10 μm and 45 μm).

## 2. Materials and Methods

To create the composites, one-component epoxy powder NEMresin1010 b (New Era Materials, Modlniczka, Poland) was used as the matrix material. Two types of graphite powder with different particle size were used as fillers: flake graphite MG1596 (Sinograf SA, Toruń, Poland) with a filler size of approximately 10 μm and MG394 (Sinograf SA, Toruń, Poland) with a size of approximately 45 μm. A series of 8 composite panels was manufactured using the hot isostatic pressing technique in accordance with the compositions included in Table 1.

**Figure 1:**
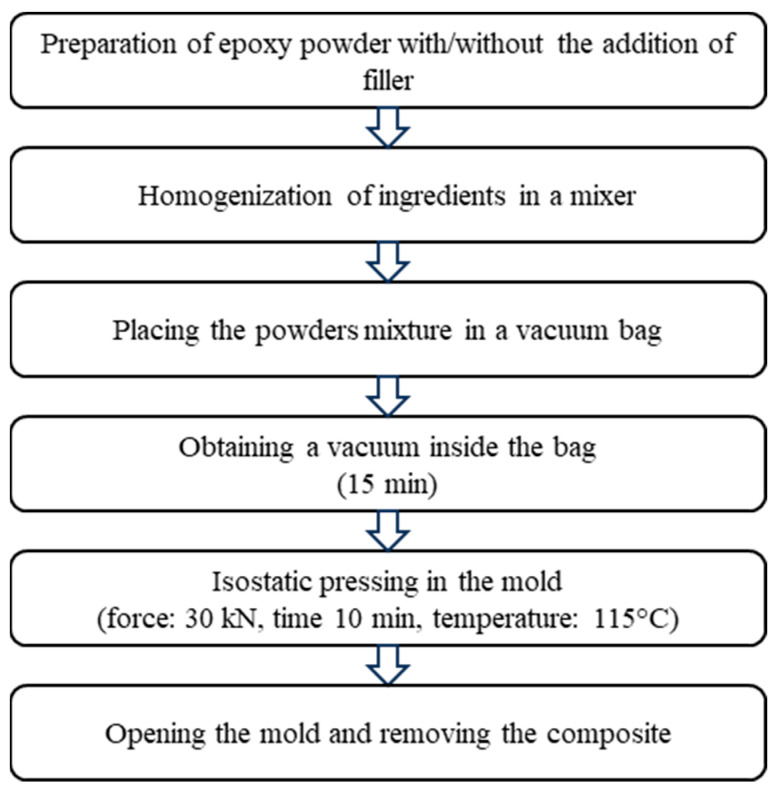
Procedure for producing composites using single-component epoxy powder with the addition of graphite particles.

The procedure for producing composites using the isostatic pressing technique is shown schematically in Figure 1. In the first stage, a mixture of epoxy powder and graphite filler was prepared using a VH type mixer (time 15 min, mixer revolutions 300 rpm, device revolutions 30 rpm, counter-rotating movement). The mixer parameters ensured homogeneous distribution of powders in the volume and no lumps of stuck resin. A portion of the epoxy powder with added graphite was placed inside a vacuum bag and then hot isostatically pressed (pressure force 30 kN, time 10 min, temperature: 115 °C). After completing the isostatic pressing, the vacuum pump was disconnected, and the mold was opened when the composite reached a temperature of 30–40 °C. In this way, flat panels with dimensions of 400 × 400 × 5 mm were obtained.

A flat panel was used for testing. Three samples were cut from each plate for tribological, hardness, glass transition temperature, and three-point bending tests. The cross-section of the material was examined using optical light microscopy on an Olympus GX71 microscope (Olympus, Tokyo, Japan). Then, using ImageJ software (version 1.54a), the percentage of the surface share of graphite particles in the composite was assessed, and the uniform distribution of particles throughout the material volume was verified.

The glass transition temperature (T_g_) was determined using the DSC technique using a DSC Q2000 machine (TA Instruments, New Castle, DE, USA). The test was carried out in the temperature range from 40 to 180 °C at a heat rate of 20 °C/min. The hardness of the samples was determined using the Brinell method for plastics using a HK460 hardness testing device (Heckert, Leipzig, Germany). The three-point bending strength was assessed in accordance with ISO 178 [41]. The test was carried out on a Shimadzu AGX-V testing machine (Shimadzu, Kyoto, Japan) with a distance between supports of 60 mm and a strain rate of 5 mm/min. Tribological tests under technically dry friction conditions were conducted using the pin-on-block method with the TM-01M tribotester [40,42]. The counterface material was a steel pin (KONTAKT S.A., Toruń, Poland) measuring 6 mm in diameter (hardened carbon steel with a hardness of 60 ± 2 HRC, DIN 100Cr6). A load of 1 MPa was applied along a path of 12 mm at a speed of 0.1 m/s, and the materials were tested over a total distance of 400 m. The obtained measurement results were summarized as function graphs showing the relationship between the friction coefficient and the distance. Using a scanning electron microscopy technique on a Hitachi S-4200 (Hitachi Group, Tokyo, Japan), friction paths were observed after tribological testing to characterize the abrasive wear mechanism and assess the potential formation of a transfer tribofilm on the material surface.

## 3. Results and Discussion

The casting of liquid epoxy resins used in previous studies allowed the introduction of 20% by weight of graphite fillers. Larger additions of graphite made it impossible to obtain a homogeneous composite, which was a limitation of the liquid two-component epoxy resin. Attempts to add more than 20% of graphite to the liquid epoxy resin resulted in the formation of a thick, non-homogeneous paste with large agglomerates of filler particles. The cross-linking process was unfavorable and led to the generation of a large amount of porosity inside the material, which was a major limitation of the casting method. The use of a single-component epoxy powder in these tests allowed the introduction of a larger amount of graphite: 30% by weight. Numerous attempts carried out to make the composite using the pressing technique allowed us to determine that inside the vacuum bag, the following layers should be placed: anti-adhesion foil, Peel Ply, a portion of epoxy powder with/without the addition of fillers, and anti-adhesion foil. The vacuum bag is then closed, and a vacuum is created inside the bag. The system is placed inside the mold and performs the isostatic pressing process while maintaining a vacuum inside the bag. After completing the pressing, normal pressure inside the bag is restored and the composite is demolded.

### 3.1. Cross-Sectional Analysis of Materials and Quantitative Evaluation

**Figure 2:**
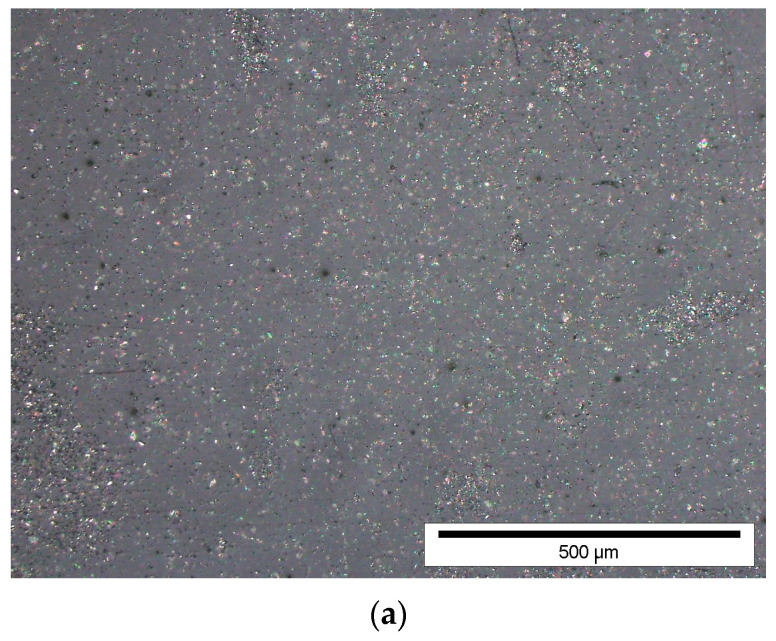

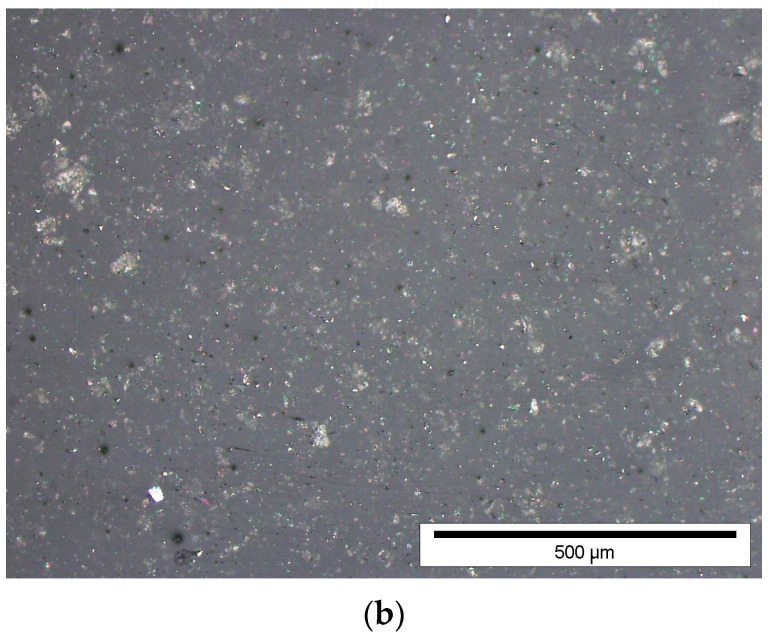
Cross-section of representative samples: (**a**) G10_30%; (**b**) G45_30% (optical microscope, 10× magnification).

Figure 2 presents images obtained using the optical light microscopy technique. One representative sample was selected for each graphite filler for a size of approximately 10 μm (Figure 2a) and for a size of approximately 45 μm (Figure 2b). In the figure, the white areas are the graphite filler, the black dots are the porosities, while the gray background represents the composite matrix. The cross-section of the material allows for a uniform distribution of the graphite filler in all samples and no tendency to agglomeration of graphite particles. In the case of casting processes with liquid epoxy resins, there are known problems with obtaining a uniform distribution of powder fillers, mainly due to sedimentation occurring under the influence of gravity [40]. The production of composites using single-component epoxy powders allows for high material homogeneity because there is no intensive movement of fillers during production. The occurrence of internal porosities whose size does not exceed 5 μm was observed. The acquired images were analyzed quantitatively to ascertain the percentage of surface coverage of graphite particles within the epoxy resin matrix. The results of quantitative image analysis are summarized in Table 2.

The surface percentage of filler particles is similar for particle sizes of 10 μm and 45 μm at the same weight content. For example, for a 10% weight addition of graphite particles sized at 10 μm, the surface coverage is 9.11%, whereas for graphite particles sized at 45 μm, the surface coverage is 8.98%; despite the same filler amount, the number of particles differs significantly. The phenomenon where the percentage of the surface area of fillers, irrespective of particle size, is similar in these studies has a beneficial impact on the analysis of the results, because one of the variables is excluded from the considerations. It is important that composites with the same weight addition of fillers and a similar surface share of graphite in the polymer matrix are compared; the only variable is the filler diameter and, therefore, the number of grains in the same mass (as the particle size decreases, their total number increases).

### 3.2. Glass Transition Temperature Examination

**Figure 3:**
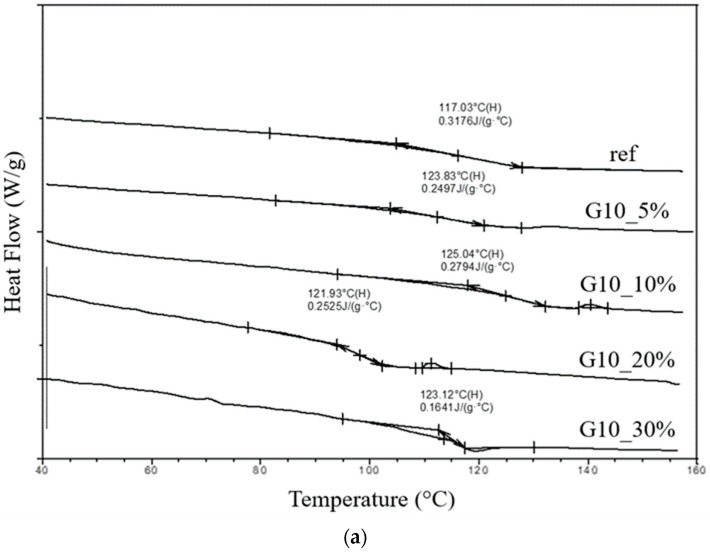

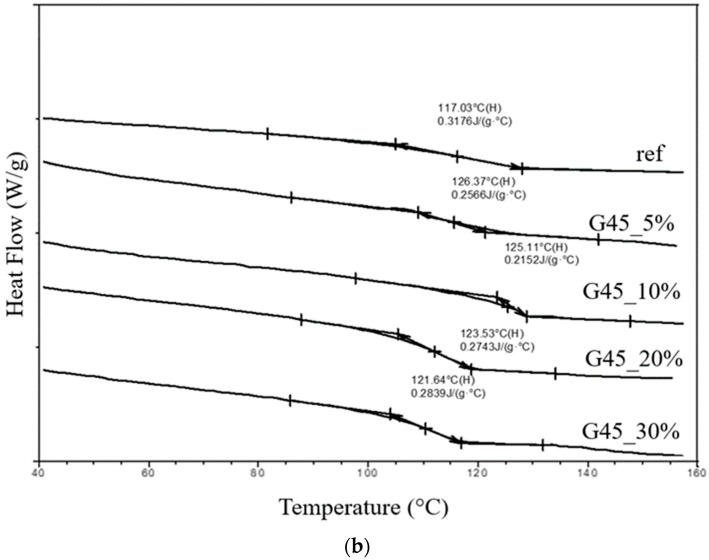
DSC curves for samples with graphite: (**a**) about 10 μm; (**b**) about 45 μm.

The results of the DSC analysis allowed for the determination of the glass transition temperature T_g_ of the composites. Determining the glass transition temperature is important and allows us to specify the thermal resistance in the actual conditions of use of the material. Exceeding the glass transition temperature may result in a loss of thermal stability of the material and affect the behavior of the material, which is hard and stiff below the T_g_ and above it becomes more flexible and viscoelastic. Moreover, materials with a high glass transition temperature are often characterized by better chemical resistance and have a longer service life. The glass transition temperatures included in Table 3 and showed in Figure 3 were determined by the DSC method using a heating rate of 20 °C/min for a temperature range of 40–180 °C. The test results indicated that the T_g_ temperature for unfilled epoxy resin is 117 °C, and the addition of fillers contributes to a rise in the glass transition temperature, which is a beneficial phenomenon. Similar behavior of the resin after adding carbon fillers was described in publication [43], where Yang showed that a well-dispersed filler in the polymer matrix contributes to an increase in T_g_ as a result of appropriate connection between resin and graphite. This interaction leads to a reduction in the mobility of the polymer chains, which leads to an increase in the glass transition temperature. Analogous results were obtained in [44], where the amount of filler to the epoxy matrix reduced the heat of reaction (ΔH) while increasing the glass transition temperature of the composite. Article [45] also reported an increase in T_g_ after adding graphite to the polyester composite matrix. A strong relationship was demonstrated between the filler surface and the matrix, where raising the surface area increases the glass transition temperature, owing to the existence of -OH and -COOH functional groups on the surface. The functional groups described limit the mobility of polymer chains in the epoxy mass. The size of the filler particles is also crucial, as introducing larger particles into the polymer may lead to more significant alterations in the glass transition temperature compared to smaller particles due to the potential restriction of polymer chain mobility by larger particles [46].

### 3.3. Hardness

**Figure 4:**
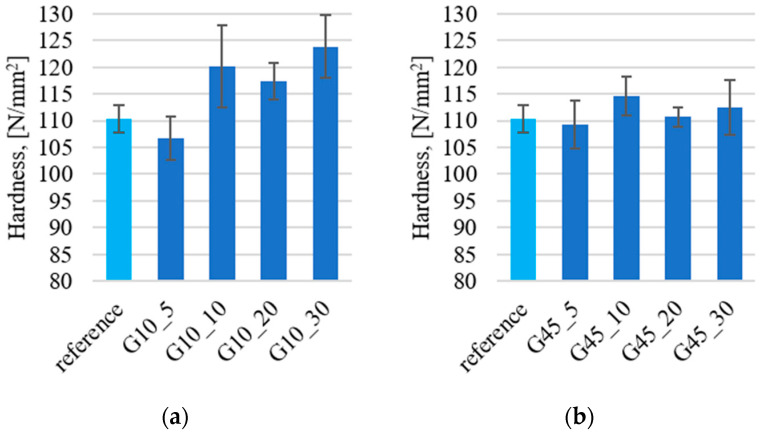
Results of Brinell hardness measurements for samples with graphite particles: (**a**) about 10 μm; (**b**) about 45 μm.

Figure 4 shows the findings from Brinell hardness measurements. The Brinell hardness test was carried out for a load of 365 N using a steel ball with a diameter of 5 mm. The unfilled epoxy resin reference sample exhibits a hardness of 110 HB. The weight addition of graphite filler above 10 μm contributes to an increase in hardness by approximately 10% at weight additions of 10–30% (Figure 4a). The observed increase in hardness after adding graphite to the polymer matrix has been described in numerous studies. The improvement in hardness after adding graphite to polyamide reported by Sathees Kumar [47] could be due to the increase in the filler surface in the matrix with the increase in the weight of graphite added and may also be the elastic response of the polymer matrix to the introduced particles. In publication [48] by ElFaham, an increase in the hardness of the rubber composite was observed after adding a graphite filler, which was related to the increased cross-linking density of the polymer after adding the filler particles. The beneficial effect of the addition of graphite to the polymer matrix of the composite was also described in publication [49] by Yang, wherein they explain that the dispersion of graphite particles increases the Young’s modulus, which contributes to obtaining higher hardness.

The addition of 5% by weight of graphite does not result in substantial changes in hardness. The addition of graphite filler size above 45 μm (Figure 4b) does not significantly increase the hardness of the material. This may confirm that too small a quantity of particles in the matrix does not contribute to changes in the polymer network, which affects the change in hardness.

### 3.4. Mechanical Properties

**Figure 5:**
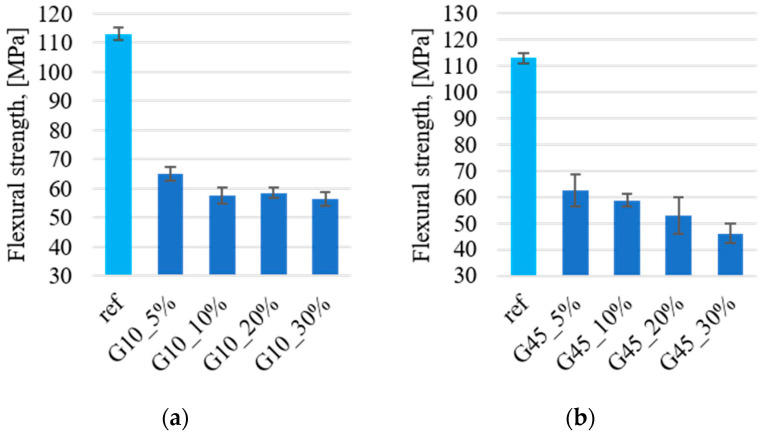
Flexural strength for composites with graphite: (**a**) about 10 μm; (**b**) about 45 μm.

The flexural strength was determined, shown in Figure 5. The results were obtained using a support spacing of 60 mm and a strain rate of 5 mm/min. Both smaller graphite particles with a size above 10 μm (Figure 5a) and larger particles with a size above 45 μm (Figure 5b) contribute to the reduction of the flexural strength. The decrease in strength is large and amounts to approximately 50% for all samples with the addition of graphite. The addition of 10 μm graphite, irrespective of the percentage addition by weight, reduces the strength from 110 MPa to approximately 60–65 MPa. In the case of graphite filler above 45 μm, a proportional decrease in flexural strength is observed in relation to the percentage of graphite. The introduction of fillers results in the formation of internal notches and porosity within the material, which further diminishes mechanical strength. In [50], González García describes similar changes in flexural strength after adding graphite filler to the polyester matrix, explaining the decrease in strength with the unfavorable effect of filler particles on the polymer cross-linking process. Similar behavior was observed and described in publication [51], where Debelak explains the decrease in the flexural strength of the composite due to the low mechanical strength and inadequate interphase connection of graphite crystallites.

### 3.5. Tribological Results

**Figure 6:**
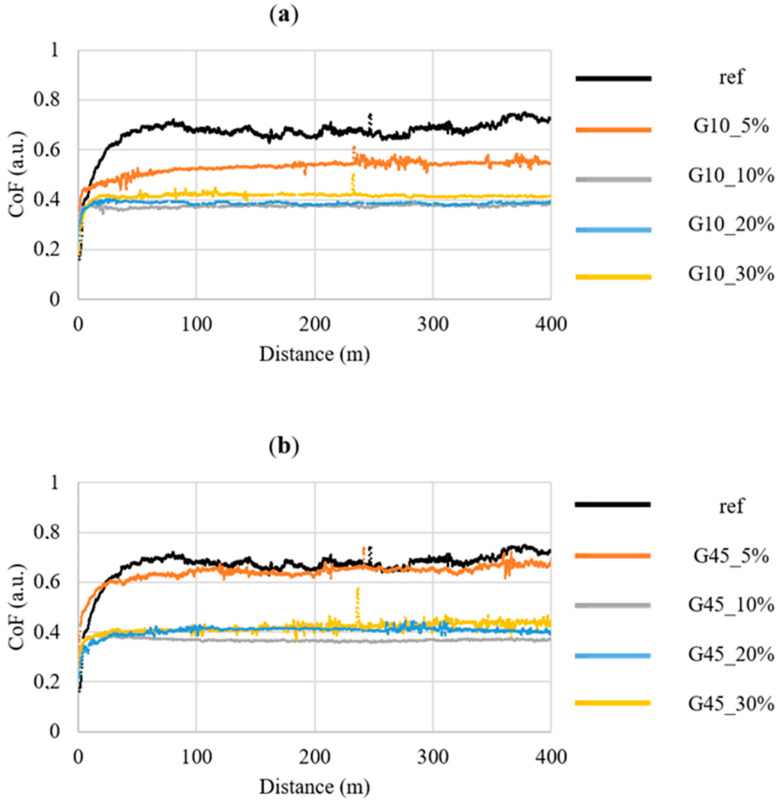
Curves of dynamic coefficient of friction vs. sliding distance for the composites containing graphite: (**a**) about 10 μm; (**b**) about 45 μm.

The focal point of this research was to ascertain the tribological properties of the composites produced with the addition of graphite filler and to describe the mechanism of tribological wear. Figure 6 and Table 4 present the results of tribological tests. The pin-on-block test results were obtained for a speed of 0.1 m/s and a load of 1 MPa, where the counterface steel pin was 6 mm in diameter. The recorded friction coefficient over a distance of 400 m for the unfilled epoxy is 0.60, which is a typical result for this type of materials tested in friction with steel. During the test, the material emitted a squeal throughout the entire distance. The friction coefficient stabilizes and goes to the plateau phase after approximately 50 m. Samples with graphite filler show a reduced coefficient of friction in comparison to the reference epoxy sample. The addition of 5% by weight of graphite fillers about 10 μm reduces the coefficient of friction by approximately 20%, while the addition of graphite about 45 μm does not lead to similar changes, and the coefficient of friction is close to the unfilled epoxy resin. The addition of 10%, 20%, and 30% by weight of fillers, irrespective of their size, results in a notable reduction in the friction coefficient by approximately 40–45%, which is advantageous. For weight additions of 10–30%, the travel distance is also shortened to approximately 20 m. It was noticed that after running in, composites with the addition of graphite do not emit squeaks and work is quiet. The decrease in the friction coefficient observed in the tests after adding graphite to the polymer matrix is typical and confirmed by other scientific reports. The authors of publication [47] observed a reduction in the coefficient of friction and abrasive wear of the composite after adding graphite to polyamide (PA 6), indicating that the most favorable parameters were achieved with a 20% addition of graphite by weight. In turn, Gilardi in article [52] described the positive impact of adding graphite on the tribological polystyrene properties; at the same time, he showed that the effect of graphite on the tribological properties of the composite is non-linear due to the fact that incorporation of graphite at weights below 10% does not significantly change the tribological properties, and the addition of 20% significantly improves the properties. In a study investigating the impact of flake graphite filler on the tribological polyetherimide properties [53], Xian demonstrated the beneficial effect of self-lubrication and limited abrasive wear and described the mechanism of creating friction films between two materials, which is crucial for reducing the friction coefficient. However, it was indicated that a 5% volume addition of graphite is insufficient for the effective creation of a transfer film between the composites and the countersample material. Analogously to the previous considerations, also in publication [54], the incorporation of graphite to the polyimide matrix has a beneficial impact on reducing the friction coefficient by approximately 40–50% and limiting the wall wear of the composite.

Test composites show limited abrasive wear after adding graphite, which is manifested by smaller mass loss and smaller volume loss in composites incorporating filler at 10–30% by weight. For the 5% weight addition, no reduced abrasive wear was observed in comparison to the reference sample of unfilled epoxy resin, which may indicate that the amount of filler is too small to provide effective protection against frictional wear. Based on the surface anylysis share of fillers in the composite structure and the obtained friction coefficients, it can be concluded that the surface share of particles is crucial for the friction coefficient, and their size in the range of 10–45 μm is not important for the tribological properties.

**Figure 7:**
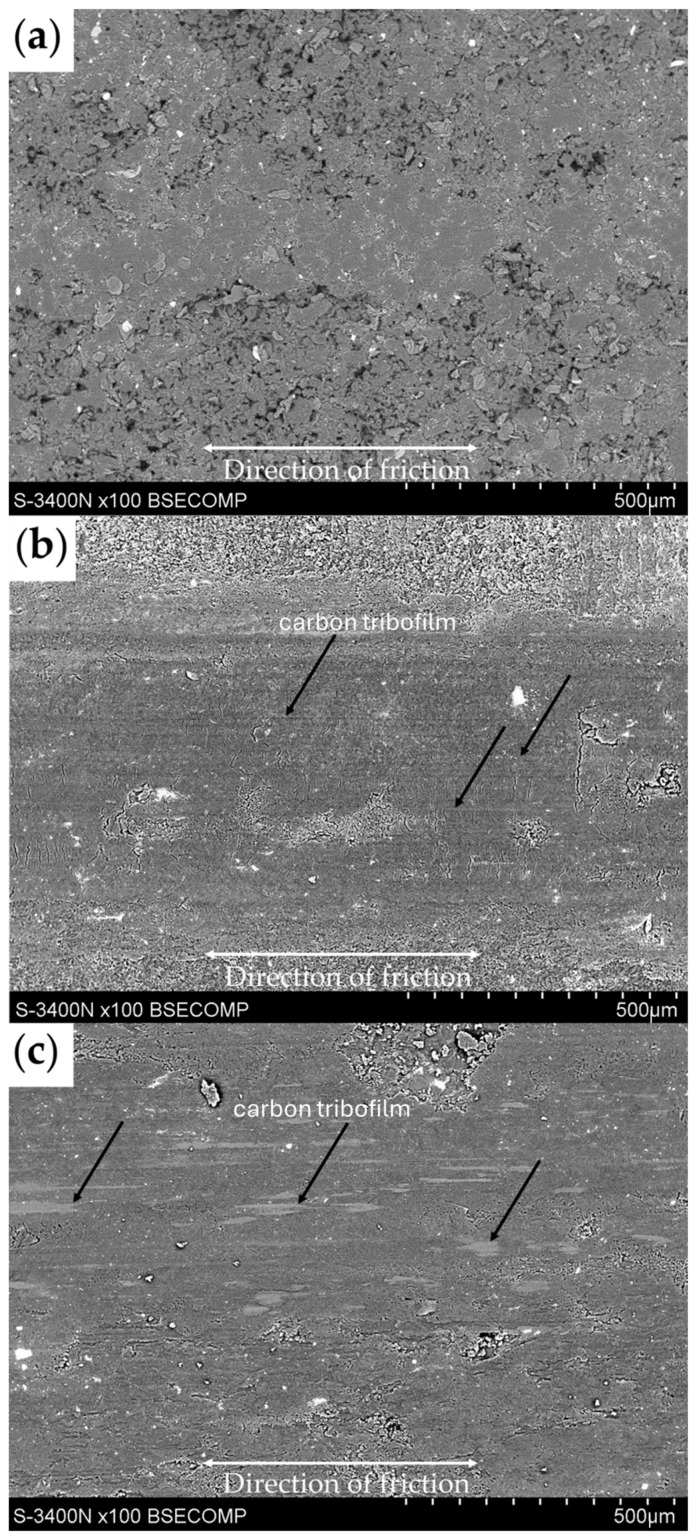
SEM images of composites after tribological testing: (**a**) reference sample; (**b**) G10_30%; (**c**) G45_30%.

The conducted research allowed us to select samples with graphite about 10% by weight as beneficial in the manufacturing of composites with self-lubricating properties due to the significantly lower coefficient of friction and abrasive wear compared to unfilled epoxy resin. The addition of graphite above 10% ensures the creation of a transfer carbon layer on the material surface as a result of friction, as shown in Figure 7, where a carbon tribofilm is clearly visible on the surface of representative samples with 30% graphite addition after the test. The development of a carbon tribofilm is essential for reducing the friction coefficient and maintaining stability beyond 20 m of sliding distance. Comparison of the obtained friction coefficients and observation of abrasion traces after the test allow us to conclude that the addition of 5% by weight of graphite fillers (with a specific surface fraction of particles of approximately 5%) is insufficient to effectively create a carbon transfer film, constituting a lubricant and ensuring self-lubricating properties. Incorporating graphite at 10–30% by weight facilitates the formation of a carbon tribofilm, ensuring effective lubrication. The phenomenon of favorable carbon tribofilm formation is known from previous literature reports. In a previous article [40], it was shown that the addition of graphite, creating a carbon tribofilm on the composite surface, leads to reduction in the friction coefficient by over 30% and protects the material against rapid wear. Myalski, in publication [55], observed that carbon reinforcement (carbon foam) acting as a solid lubricant allows for the effective formation of a carbon tribofilm.

**Figure 8:**
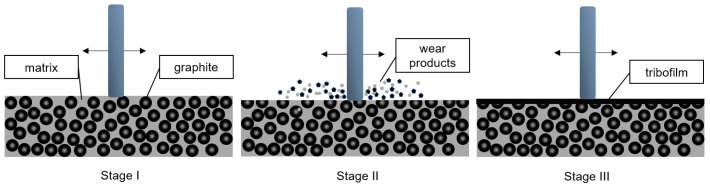
Scheme of the creation of a carbon tribofilm on the composite surface.

The observations of the behavior of materials during friction allowed us to confirm the model of the creation of a transfer film. It was proven that the friction behavior of the composite with graphite addition exhibits a three-stage nature, as shown in Figure 8. In the first stage, the counter-sample material contacts the composite, and both materials move in a back-and-forth motion relative to each other. In the second stage, the mutual movement of materials leads to the wear of the composite, and there is a gradual loss of the filler and matrix as a result of shearing of irregularities. At this stage, the friction coefficient changes and increases. In the third stage, after the formation of the tribofilm, the friction coefficient stabilizes, and further abrasive wear is reduced to a minimum.

## 4. Conclusions

The conducted research allowed us to draw the following conclusions:

- single-component epoxy powders allow the introduction of more graphite fillers than in the case of formerly investigated liquid resins—in liquid epoxy resin, the addition of 20% by weight of graphite was the limit;
- single-component epoxy powders allow manufacturing products at shorter time periods in comparison with the liquid resins, which is a big advantage when implementing the technology for large-scale production;
- the addition of graphite increases the glass transition temperature (T_g_). The unfilled epoxy matrix shows T_g_ at 117 °C, whilst the composites with graphite show T_g_ at average 122–127 °C, which probably results from the limited mobility of polymer chains after the introduction of the graphite filler;
- graphite with a particle size of 10 μm leads to an increase in the hardness of the composite, while particles with a size of 45 μm do not lead to similar changes, which is largely due to the lesser number of particles in the same mass, which affects the interaction between the matrix and the filler;
- the addition of graphite, irrespective of the weight fraction and particle size, impacts the flexural strength, leading to a reduction of approximately 50%;
- the addition of graphite at 10, 20, 30% by weight leads to a reduction in the friction coefficient by approximately 40–45% and a reduction in abrasive wear, which results from the effective formation of transfer film on the material;
- 5% weight addition of graphite is insufficient for the effective creation of transfer film, which is manifested by a high friction coefficient and significant abrasive wear;
- the manufactured composites with a graphite filler weight addition of 10–30% have properties typical of self-lubricating materials, where graphite acts as a solid lubricant. These materials can be effectively used in the manufacturing of self-lubricating plain bearings, friction tracks, or bearing shells.

## Figures and Tables

**Table 1:** Compositions of samples with designations.

Sample	Weight Addition, [%]	
	Graphite < 10 μm	Graphite < 45 μm
reference ^1^	0	0
G10_5%	5	0
G10_10%	10	0
G10_20%	20	0
G10_30%	30	0
G45_5%	0	5
G45_10%	0	10
G45_20%	0	20
G45_30%	0	30

^1^ Unfilled cured epoxy powder.

**Table 2:** Quantitative share of graphite fillers in the cross-section of the composite.

Sample	Area Coverage, [%]
reference	0.00
G10_5	5.13
G10_10	9.11
G10_20	15.52
G10_30	20.61
G45_5	5.44
G45_10	8.98
G45_20	16.35
G45_30	21.01

**Table 3:** Glass transition temperatures.

Sample	Glass Transition Temperature, [°C]
reference	117.03
G10_5	123.83
G10_10	125.04
G10_20	121.93
G10_30	127.54
G45_5	126.37
G45_10	125.11
G45_20	123.53
G45_30	121.64

**Table 4:** Dynamic coefficient of friction and volume loss of the tested composite materials.

Sample	Dynamic Coefficient of Friction, μ	Standard Deviation of μ	Volume Loss, [cm^3^]	Mass Loss, [g]
reference	0.67	0.03	0.0007	0.0008
G10_5%	0.54	0.02	0.0006	0.0007
G10_10%	0.38	0.01	0.0002	0.0002
G10_20%	0.42	0.02	0.0003	0.0003
G10_30%	0.39	0.02	0.0004	0.0004
G45_5%	0.65	0.03	0.0011	0.0013
G45_10%	0.37	0.01	0.0002	0.0002
G45_20%	0.42	0.02	0.0005	0.0006
G45_30%	0.41	0.02	0.0006	0.0007

## Data Availability

The original contributions presented in the study are included in the article, further inquiries can be directed to the corresponding author.
